# Effects of inositol and alpha lipoic acid combination for polycystic ovary syndrome: A protocol for systematic review and meta-analysis

**DOI:** 10.1097/MD.0000000000020696

**Published:** 2020-07-24

**Authors:** Wenwen Lei, Yang Gao, Shiruo Hu, Dongying Liu, Qiu Chen

**Affiliations:** Hospital of Chengdu University of Traditional Chinese Medicine, PR China.

**Keywords:** alpha lipoic acid, inositol, meta-analysis, polycystic ovary syndrome, protocol, systematic review

## Abstract

**Background:**

Polycystic ovary syndrome (PCOS), an intricate and multifactorial disease, has characteristics of diverse clinical, metabolic and endocrine disorder. It represents a primary cause of infertility in reproductive women, which seriously affects the physical and mental health of patients. Several small studies have indicated that inositol and alpha lipoic acid (ALA) supplementation can ameliorate the outcomes in terms of menstrual cyclicity, ovulation and hyperinsulinemia in PCOS women. However, there is a lack of sufficient evidence to affirm this practice. Consequently, we aim to objectively review and estimate the efficacy and safety of inositol plus ALA in adult women suffering from PCOS.

**Methods and analysis:**

We will retrieve PubMed, EMBASE, The Web of Science, The Cochrane Library of Controlled Trials, Clinical Trials.gov, Chinese Biomedical Literature Database (CBM), China National Knowledge Infrastructure (CNKI), Chinese Scientific Journal Database (VIP database), Wan-Fang database with no specific limitations on language. Simultaneously we will manually retrieve reference lists and grey literature to acquire potential eligibility. We will restrict our search to randomized controlled trials (RCTs) of inositol in combination with ALA for PCOS. Researchers will separately identify studies, extract data and evaluate the quality of studies. We will conduct risk of bias estimates, data synthesis and analysis using Review Manager 5.3 software.

**Results and conclusion:**

The study will comprehensively determine the effectiveness and safety of inositol conjunct with ALA therapy for PCOS. Meanwhile we intend to disseminate the final findings in a peer-reviewed journal to help patients, clinicians and health policymakers select treatment strategy of PCOS by providing high-quality evidence.

## 1 Introduction

Polycystic ovary syndrome (PCOS), one of the most common endocrine dysfunction in reproductive-age women, affects approximately 5% to 21% premenopausal women under the different diagnostic criteria.^[1]^ In the light of the Rotterdam diagnostic criteria, PCOS represents a multifactorial condition including at least 2 of the 3 clinical characteristics: chronic intermittent or absent ovulation, clinical or biochemical hyperandrogenism and polycystic ovaries morphology on ultrasound.^[2]^ It frequently has the features of anovulatory infertility, obesity, type 2 diabetes and cardiometabolic dysfunction.^[3,4]^ Apart from this, PCOS has a detrimental effect on both physical health and mental wellbeing. Compared with the general population, women diagnosed with PCOS have been reported higher rates of anxiety and depression.^[5]^

Although the exact pathogenetic mechanism of PCOS remains unclear due to its heterogeneous nature, some risk factors and vital triggers have been clarified. Among them insulin resistance (IR) has a pivotal role to play during the onset of PCOS, and its prevalence amounts to 70%.^[6,7]^ IR and accompanying hyperinsulinemia accelerate the production of ovarian androgen while decreasing hepatic sex hormone binding globulin, exacerbating hyperandrogenism.^[8]^ Aside from IR, oxidative stress (OS) might further underpin PCOS and its metabolic complications.^[9]^ It is reported to be connected with central obesity, hypertension, impaired glucose, IR and dyslipidemia.^[10,11]^ Hyperandrogenism possibly occurs on account of the inflammatory response of abnormal follicular membrane cells in the ovary to oxygen free radicals.^[12]^

Given the crucial roles of IR and OS in the pathological mechanisms of PCOS, inositol plus alpha lipoic acid (ALA) combination is a promising therapeutic approach with none significantly detrimental effects for PCOS women.^[13]^ Inositol is a nutraceutical compound (a sugar alcohol) with nine stereoisomers, and in particularly myo-inositol (MI) and D-chiro-inositol (DCI) are the 2 most common isoforms in tissue. Both seem to be able to improve metabolism and endocrine parameters, acting as insulin sensitizers and antioxidant.^[14–16]^ ALA represents another potent free radical scavengers and natural cofactor of mitochondrial dehydrogenase complexes. When administered alone or in combination with inositol, it is beneficial to the amelioration of glucose control, IR, metabolic and endocrine disturbances in PCOS sufferers.^[17–20]^

More recently, a new therapeutic strategy of inositol in conjunction with ALA has been proposed as it appears to exist a synergistic efficacy to insulin sensitivity and OS for PCOS. Some clinical studies reported that this combination therapy had a positive effect with few adverse events.^[21–23]^ However, due to the limited sample sizes in majority of clinical trials, there is a lack of high-quality evaluations of benefits and harm of their combination. Therefore, we plan to systematically appraise the effectiveness and safety of the inositol plus ALA combination therapy in women suffering from PCOS by conducting a meta-analysis on the basis of existent studies, anticipating providing reliable evidence for clinical practice.

## 2 Methods

### 2.1 Study registration

This protocol of the systematic review and meta-analysis is registered at the International Platform of Registered Systematic Review and Meta-analysis Protocols (INPLASY) with number INPLASY202050011, and includes a prespecified analytical plan which is available in full on the inplasy.com (https://doi.org/10.37766/inplasy2020.5.0011). It is structured in accordance with the preferred reporting items for systematic reviews and meta-analyses protocols (PRISMA-P) statement guidelines and the Cochrane handbook for systematic reviews of interventions.^[24–26]^

### 2.2 Inclusion criteria

#### 2.2.1 Types of studies

Just published and ongoing randomized controlled trials (RCTs) and human (women) studies are eligible for inclusion, with no restrictions imposed on the method of randomization or blinding used, or the language and date of publication. However, reviews, case series, animal and vitro investigations and non-randomized controlled trials will be excluded. Priority will be given to the report with the largest sample size, if a study with the same population is repetitively published.

#### 2.2.2 Types of participants

Adult women diagnosed as PCOS will be incorporated, in accordance with Rotterdam Criteria 2003, National Institute of Health Criteria 2012, or Androgen Excess and PCOS 2009, regardless of ethnicity. Other etiologies of menstrual disturbance and hyperandrogenism will be eliminated, for instance congenital adrenal hyperplasia, androgen-secreting tumors, Cushing's syndrome. Adolescents (under 18 years old) and post-menopausal women (more than 50 years old) will be excluded from the review.

#### 2.2.3 Types of interventions and controls

Intervention strategies comprise inositol (MI or DCI) and ALA combination, with no restrictions on dosage, frequency and duration. While controls apply for blanks, placebo, any active treatment or lifestyle interventions such as diet and exercise.

#### 2.2.4 Types of outcome measures

##### 2.2.4.1 Primary outcomes

Main effect outcomes are mainly composed of menstrual cycle regulation, body mass index (BMI), homeostasis model assessment of insulin resistance (HOMA-IR).

##### 2.2.4.2 Secondary outcomes

Other potentially important outcome parameters involve the following aspects:

(1) Clinical outcomes: Ferriman–Gallway score, waist to hip ratio (WHR), systolic and diastolic blood pressure.
(2) Metabolic outcomes: blood glucose and insulin parameters, total cholesterol (TC), triglycerides (TG), high-density lipoprotein (HDL) and low-density lipoprotein (LDL).
(3) Endocrine outcomes: leutinizing hormone (LH), follicle stimulating hormone (FSH), testosterone, estradiol (E2), progesterone, serum sex hormone binding globulin (SHBG) and dehydroepiandrosterone sulphate (DHEAS).
(4) Any adverse event.

### 2.3 Search methods

We will carry out a comprehensive retrieval in these electronic bibliographic databases from inception until May 2021: PubMed, EMBASE, The Web of Science, The Cochrane Library of Controlled Trials, Clinical Trials.gov, Chinese Biomedical Literature Database (CMB), China National Knowledge Infrastructure (CNKI), Chinese Scientific Journal Database (VIP database), Wan-Fang database with no restrictions on the language. At the same time, we will hand-search reference lists and grey literature to obtain additional citations.

We will apply a search method combining MeSH terms and free words. Search terms will be as follows: PCOS, inositol, MI, DCI, ALA, randomized controlled trials, etc. We will adjust retrieval methods on the basis of diverse electronic databases. Before the final analyses, we will search again to further identify eligible studies. The full search process on PubMed is provided in the following Table 1.

**Table 1:**
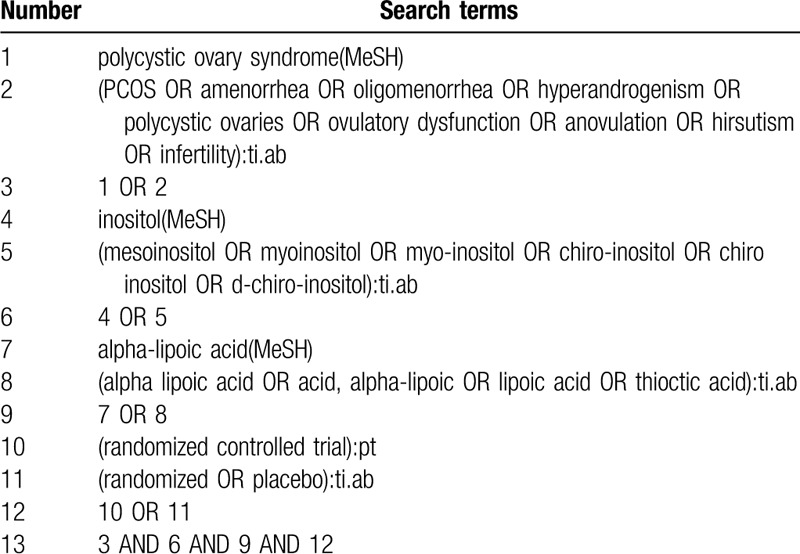
Search strategy for PubMed.

### 2.4 Data collection and analysis

#### 2.4.1 Studies selection

**Figure 1:**
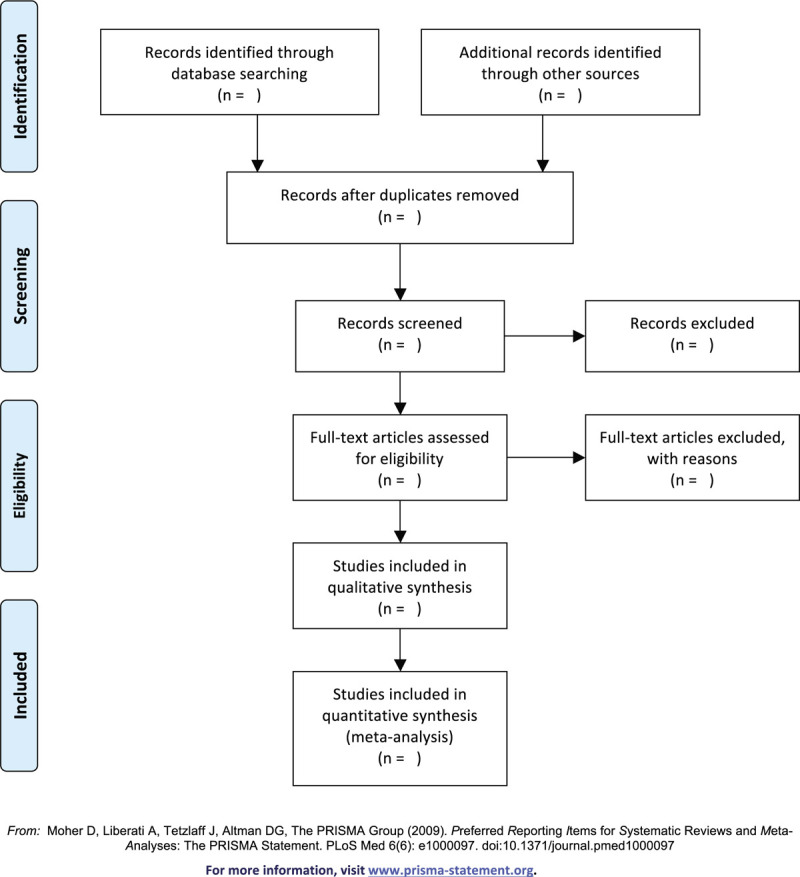
Flow diagram of study selection process.

Two methodological trained researchers (WL and SH) will separately select the pertinent literature. Retrieved literature will be imported into the software of Endnote X9 and duplicate literature will be deleted. Then, we will screen the titles and abstracts of citations for compliance with the inclusion and exclusion criteria. Reserved articles will be ulteriorly estimated for inclusion through the full texts of articles. Ultimately, the results will be cross-checked repeatedly by reviewers. Any discrepancy will be settled by discussion or negotiation with a superior researcher (YG). In addition, the selection procedure will be recorded adopting a PRASMA flow chart (Fig. 1).

#### 2.4.2 Data extraction and management

Two investigators (WL and DL) will abstract data into Microsoft Excel spreadsheet independently. These data will be extracted from eligible studies: study characteristics (first author, publication time, country, sample size, study design and duration, etc), participant information (age, sex, diagnostic criteria for PCOS, etc), intervention details (type of intervention and control, dosage, frequency, administration route, course of treatment, etc), outcome indicators for safety and effectiveness. In consideration of cautiousness, two investigators will finally cross-check the above information. Any divergence will be settled by mutual discussion or consulting with another investigator (QC). We will try to get in touch with the corresponding author to acquire additional detailed materials where complete data are unavailable.

#### 2.4.3 Risk of bias assessment

Two independent reviewers (SH and DL) will appraise the quality of each research method by employing the Cochrane risk of bias assessment tool. There are seven components affiliated with bias risk, including randomized method, allocation concealment, blinding of participants and staff, blinding of outcome assessment, completeness of outcome data, selective reporting and other underlying biases. Each entry will be allocated a RoB rating of low, high or unclear risk. Diverse opinions between both reviewers over certain studies will get a consensus or arbitration with a third one (YG) as required.

#### 2.4.4 Measures of treatment effect

Pooled methods are diverse based on the types of variables. We will adopt risk ratio (RR) in order to summarize the effect size of each dichotomous variable. While for each continuous variable, the mean difference will be analyzed. We will present 95% confidence intervals for all outcomes.

#### 2.4.5 Management of missing data

We will get into contact with the first author or correspondent author of relevant studies to obtain the complete and sufficient information via email or telephone. We will exclude the study where the complete and informative data is not available. Meanwhile, we will perform a sensitivity analysis to investigate the possible influence of absent data on the overall results of the systematic review.

#### 2.4.6 Assessment of heterogeneity

We will examine heterogeneity between inclusions using Cochran Q and I^2^ statistic.^[27]^ In the case of *P* > .10 and I^2^ < 50%, the variables are regarded as be homogeneous, and we will apply the fixed-effect models. Conversely, the random-effect models will be adopted if substantial heterogeneity exist. If the heterogeneity is considerable, we will carry out a subgroup analysis to clarify possible causes of statistical inconsistency.

#### 2.4.7 Assessment of reporting biases

Reporting biases will be estimated by means of funnel plot and Egger's regression test if there are 10 or more eligible trials.^[28]^ Supposing that funnel plot is asymmetric, the potential sources for the asymmetry will be analysed.

#### 2.4.8 Data synthesis and analysis

We will synthesize and analyze data with the software of Review Manager version 5.3 offered by the Cochrane Collaboration. In addition, we will merge data as mean difference or RR with 95% confidence intervals for continuous variables or the categorical, respectively. Homogeneous data will be pooled through a fixed effect model. Inversely, a random effect model will be selected. We will implement subgroup analysis or sensitivity analysis to detect the causes if there exists prominent heterogeneity. We will present a narrative review of study results where the meta-analysis is not appropriate.

#### 2.4.9 Subgroup analysis

To identify explanations of heterogeneity between adequate studies (>10) and answer questions of clinical interest, we will conduct subgroup analyses within the following aspects, type of inositols, family history of diabetes mellitus and IR.

#### 2.4.10 Sensitivity analysis

We plan to verify the stability and reliability of the review conclusions with the method of sensitivity analysis for the primary outcomes. We will eliminate each of the eligible studies one by one, and then merge the data and re-analyze. Subsequently, the distinction between the regenerated effects and the original effects will be compared. At the same time, we will determine the impact of certain factors on the conclusions of meta-analysis, such as risk of bias, effect sizes, statistical models.

#### 2.4.11 Grading the quality of evidence

We will rate overall certainty in the cumulative evidence for included trials on the basis of the Grading of Recommendations Assessment, Development and Evaluation (GRADE) instrument^[29]^ from five considerations, including study limitations, consistency of effect, indirectness, imprecision, and publication bias. The whole quality and strength of evidence will be precisely categorized into high, moderate, low and very low to achieve transparency.^[30,31]^

#### 2.4.12 Ethics and dissemination

This protocol receives no ethical approval because it is for a meta-analysis and all the analyses are based on previously published data. We will publish the final conclusions of our study in an open access academic journal electronically and in print.

## 3 Discussion

As a heterogeneous clinical condition, PCOS adversely affects endocrine, metabolic and psychological health of reproductive-aged women. It exhibits several phenotypes reflecting differences in pathophysiological processes. Routine treatments for PCOS include lifestyle management and pharmacotherapy consisting of oral contraceptive pill (OCP), insulin sensitizers, cyclic progestins, anti-androgens and fertility treatments. However, these conventional therapies are deficient. In recent years, some trials have reported that inositol and ALA combination is likely to act as a promising and safe therapy for PCOS women via improving IR and oxidative stress status.^[21–23]^ Therefore, with a systematic review and meta-analysis, we are aimed at identifying the effectiveness and certainty of evidence for clinical application of inositol combined with ALA therapy for PCOS. Simultaneously, we anticipate this study will help patients and practitioners make the better decision in the treatment of PCOS.

## Author contributions

**Conceptualization:** Wenwen Lei, Yang Gao, Qiu Chen.

**Data curation:** Wenwen Lei, Shiruo Hu.

**Formal analysis:** Wenwen Lei, Yang Gao.

**Funding acquisition:** Qiu Chen.

**Investigation:** Shiruo Hu, Dongying Liu.

**Methodology:** Wenwen Lei, Yang Gao, Qiu Chen.

**Writing – original draft:** Wenwen Lei.

**Writing – review & editing:** Wenwen Lei, Yang Gao, Qiu Chen.

## References

[1] Lizneva D Suturina L Walker W Criteria, prevalence, and phenotypes of polycystic ovary syndrome. Fertil Steril 2016;106:6–15. 27233760 10.1016/j.fertnstert.2016.05.003

[2] The Rotterdam ESHRE/ASRM sponsored PCOS consensus workshop group. Revised 2003 consensus on diagnostic criteria and long-term health risks related to polycystic ovary syndrome (PCOS). Hum Reprod 2004;19:41–7. 14688154 10.1093/humrep/deh098

[3] Moran LJ Misso ML Wild RA Impaired glucose tolerance, type 2 diabetes and metabolic syndrome in polycystic ovary syndrome: a systematic review and meta-analysis. Hum Reprod Update 2010;16:347–63. 20159883 10.1093/humupd/dmq001

[4] Teede H Deeks A Moran L Polycystic ovary syndrome: a complex condition with psychological, reproductive and metabolic manifestations that impacts on health across the lifespan. BMC Med 2010;8:41. 20591140 10.1186/1741-7015-8-41 PMC2909929

[5] Cinar N Kizilarslanoglu MC Harmanci A Depression, anxiety and cardiometabolic risk in polycystic ovary syndrome. Hum Reprod 2011;26:3339–45. 21984577 10.1093/humrep/der338

[6] Diamanti-Kandarakis E Dunaif A Insulin resistance and the polycystic ovary syndrome revisited: an update on mechanisms and implications. Endocr Rev 2012;33:981–1030. 23065822 10.1210/er.2011-1034 PMC5393155

[7] Carmina E Lobo RA Use of fasting blood to assess the prevalence of insulin resistance in women with polycystic ovary syndrome. Fertil Steril 2004;82:661–5. 15374711 10.1016/j.fertnstert.2004.01.041

[8] Greenwood EA Huddleston HG Insulin resistance in polycystic ovary syndrome: concept versus cutoff. Fertil Steril 2019;112:827–8. 31731944 10.1016/j.fertnstert.2019.08.100

[9] Escobar-Morreale HF Luque-Ramirez M San Millan JL The molecular-genetic basis of functional hyperandrogenism and the polycystic ovary syndrome. Endocr Rev 2005;26:251–82. 15561799 10.1210/er.2004-0004

[10] Sabuncu T Vural H Harma M Oxidative stress in polycystic ovary syndrome and its contribution to the risk of cardiovascular disease. Clin Biochem 2001;34:407–13. 11522279 10.1016/s0009-9120(01)00245-4

[11] Patel S Polycystic ovary syndrome (PCOS), an inflammatory, systemic, lifestyle endocrinopathy. J Steroid Biochem Mol Biol 2018;182:27–36. 29678491 10.1016/j.jsbmb.2018.04.008

[12] Gonzalez F Rote NS Minium J Reactive oxygen species-induced oxidative stress in the development of insulin resistance and hyperandrogenism in polycystic ovary syndrome. J Clin Endocrinol Metab 2006;91:336–40. 16249279 10.1210/jc.2005-1696

[13] El Hayek S Bitar L Hamdar LH Poly cystic ovarian syndrome: an updated overview. Front Physiol 2016;7:124. 27092084 10.3389/fphys.2016.00124 PMC4820451

[14] Unfer V Porcaro G Updates on the myo-inositol plus D-chiro-inositol combined therapy in polycystic ovary syndrome. Expert Rev Clin Pharmacol 2014;7:623–31. 24898153 10.1586/17512433.2014.925795

[15] Nestler JE Unfer V Reflections on inositol(s) for PCOS therapy: steps toward success. Gynecol Endocrinol 2015;31:501–5. 26177098 10.3109/09513590.2015.1054802

[16] Thomas MP Mills SJ Potter BV The other” inositols and their phosphates: synthesis, biology, and medicine (with recent advances in myo-inositol chemistry). Angew Chem Int Ed Engl 2016;55:1614–50. 26694856 10.1002/anie.201502227 PMC5156312

[17] Masharani U Gjerde C Evans JL Effects of controlled-release alpha lipoic acid in lean, nondiabetic patients with polycystic ovary syndrome. J Diabetes Sci Technol 2010;4:359–64. 20307398 10.1177/193229681000400218 PMC2864173

[18] Di Tucci C Di Feliciantonio M Vena F Alpha lipoic acid in obstetrics and gynecology. Gynecol Endocrinol 2018;34:729–33. 29726290 10.1080/09513590.2018.1462320

[19] Genazzani AD Shefer K Della Casa D Modulatory effects of alpha-lipoic acid (ALA) administration on insulin sensitivity in obese PCOS patients. J Endocrinol Invest 2018;41:583–90. 29090431 10.1007/s40618-017-0782-z

[20] Fruzzetti F Capozzi A Canu A Treatment with d-chiro-inositol and alpha lipoic acid in the management of polycystic ovary syndrome. Gynecol Endocrinol 2019;35:506–10. 30612488 10.1080/09513590.2018.1540573

[21] Cianci A Panella M Fichera M d-chiro-Inositol and alpha lipoic acid treatment of metabolic and menses disorders in women with PCOS. Gynecol Endocrinol 2015;31:483–6. 25893270 10.3109/09513590.2015.1014784

[22] De Cicco S Immediata V Romualdi D Myoinositol combined with alpha-lipoic acid may improve the clinical and endocrine features of polycystic ovary syndrome through an insulin-independent action. Gynecol Endocrinol 2017;33:698–701. 28434274 10.1080/09513590.2017.1313972

[23] Artini PG Obino MER Micelli E Effect of d-chiro-inositol and alpha-lipoic acid combination on COH outcomes in overweight/obese PCOS women. Gynecol Endocrinol 2020;1–5. doi:10.1080/09513590.2020.1737007. 10.1080/09513590.2020.1737007 32157927

[24] Shamseer L Moher D Clarke M Preferred reporting items for systematic review and meta-analysis protocols (PRISMA-P) 2015: elaboration and explanation. BMJ 2015;350:g7647. 25555855 10.1136/bmj.g7647

[25] Moher D Shamseer L Clarke M Preferred reporting items for systematic review and meta-analysis protocols (PRISMA-P) 2015 statement. Syst Rev 2015;4:1. 25554246 10.1186/2046-4053-4-1 PMC4320440

[26] Cumpston M Li T Page MJ Updated guidance for trusted systematic reviews: a new edition of the Cochrane Handbook for Systematic Reviews of Interventions. Cochrane Database Syst Rev 2019;10:ED000142 doi:10.1002/14651858.ED000142. 31643080 10.1002/14651858.ED000142 PMC10284251

[27] Higgins JP Thompson SG Quantifying heterogeneity in a meta-analysis. Stat Med 2002;21:1539–58. 12111919 10.1002/sim.1186

[28] Murad MH Chu H Lin L The effect of publication bias magnitude and direction on the certainty in evidence. BMJ Evid Based Med 2018;23:84–6. 10.1136/bmjebm-2018-110891 PMC5969367 29650725

[29] Andrews J Guyatt G Oxman AD GRADE guidelines: 14. Going from evidence to recommendations: the significance and presentation of recommendations. J Clin Epidemiol 2013;66:719–25. 23312392 10.1016/j.jclinepi.2012.03.013

[30] Atkins D Best D Briss PA Grading quality of evidence and strength of recommendations. BMJ 2004;328:1490. 15205295 10.1136/bmj.328.7454.1490 PMC428525

[31] Guyatt GH Oxman AD Vist GE GRADE: an emerging consensus on rating quality of evidence and strength of recommendations. BMJ 2008;336:924–6. 18436948 10.1136/bmj.39489.470347.AD PMC2335261

